# A vital role for Angptl3 in the PAN-induced podocyte loss by affecting detachment and apoptosis in vitro

**DOI:** 10.1186/s12882-015-0034-4

**Published:** 2015-03-29

**Authors:** Rufeng Dai, Yi Lin, Haimei Liu, Jia Rao, Yihui Zhai, Xiliang Zha, Xiaoyan Fang, Hong Xu

**Affiliations:** Department of Nephrology and Rheumatism, Children’s Hospital of Fudan University, Shanghai, 201102 China; Department of Pediatrics, Affiliated Hospital of Qingdao University, Shandong, 266003 China; Department of Biochemistry and Molecular Biology, Shanghai Medical College, Fudan University, Shanghai, 200032 China

**Keywords:** Angptl3, Podocyte loss, F-actin rearrangement, Detachment, Apoptosis

## Abstract

**Background:**

Podocyte detachment and apoptosis are two risk factors causing podocyte loss, F-actin rearrangement is involved in detachment and apoptosis. However, the nature of events that promote detachment and apoptosis of podocytes and whether detachment occurred simultaneously with apoptosis are still unclear. Previously, it was found that angiopoietin-like3 (Angptl3) induces F-actin rearrangement in podocytes. In this study we investigate whether Angptl3 influences podocyte loss (detachment and apoptosis) and the process through which Angptl3 exactly influenced the podocyte loss.

**Methods:**

In conditionally immortalized mice podocytes, recombinant mice Angptl3 protein (rm-Angptl3) was used to mimic Angptl3 overexpression model and transfection with small interfering RNA (siRNA) to knockdown the expression of Angptl3. Both flow cytometry analysis and terminal deoxynucleotidyl transferase-mediated dUTP nick-end labeling assay were used to detect apoptosis. Rearrangement of F-actin was assessed using confocal microscopy. Western blot assay was used to measure levels of Angptl3, integrin α3β1, integrin-linked kinase (ILK), p53, caspase 3, and phosphorylation of integrin β1.

**Results:**

In a puromycin aminonucleoside (PAN)-induced podocyte injury model, rm-Angptl3 accelerated the loss of podocytes, both detachment and apoptosis occurred, and F-actin rearrangement is involved in the process. However, knockdown of Angptl3 by siRNA markedly ameliorated these injuries. Observed effects were partially correlated with the altered integrin α3β1, ILK and p53, rather than caspase 3.

**Conclusions:**

Angptl3 is a novel factor involved in the PAN-induced podocyte loss by affecting detachment and apoptosis in vitro. This study helps to deepen the understanding of the mechanisms of podocyte loss and lays the foundation for developing a new successful therapy for podocyte injury via lower expression of Angptl3.

## Background

Podocytes are terminally differentiated and highly specialized cells that reside on the glomerular basement membrane (GBM). As crucial components of the glomerular filtration barrier, podocytes play a key role in the pathogenesis of glomerular diseases [1]. Due to limited podocytic proliferative capacity, it has been suggested that podocytes that are lost cannot be effectively replaced, causing damage to glomerular filtration barrier, development of proteinuria and progressive loss of renal function [2-5].

Angiopoietin-like-3 (Angptl3), a secreted protein, is mainly expressed in hepatocytes and weakly expressed in normal kidney cells [6]. Our previous study found that the expression of Angptl3 was upregulated in nephrotic syndrome kidney tissue, and altered expression of Angptl3 in the glomerulus was associated with proteinuria and foot process effacement in kidney diseases [7,8]. Moreover, it was confirmed that following treatment with adriamycin or puromycin aminonucleoside (PAN), the expression of Angptl3 in podocytes was especially upregulated, and Angptl3 was involved in podocyte injury, Angptl3 increased the motility of podocytes, and ameliorated the PAN-induced detachment of podocytes [9,10]. Recently, it was further confirmed that Angptl3 induces F-actin rearrangement [11]. Podocyte loss is caused by detachment of cells from the GBM and/or by apoptosis (programmed cell death) [12]. Seminal studies have shown that podocyte cytoskeletal disaggregation preceded, and detachment from the GBM occurred in the PAN model; actin filament played a role in the regulation of cellular apoptosis, and disruption of F-actin could lead to increased levels of apoptosis [13,14]. Whether Angptl3 influences podocyte loss by affecting detachment and apoptosis is a crucial question that warrants further study.

Podocytes adhere to the GBM principally via integrin α3β1, which is believed to be a major receptor for extracellular matrix components of the GBM [15]. Studies have reported that lower expression of integrin α3β1 in podocytes is found in a range of glomerular disorders in humans, and in a variety of experimental animal models of renal disease [16]. Integrin-linked kinase (ILK) plays a key role in integrin-mediated cell adhesion and signaling [17]. Overexpression of ILK in podocytes might reduce matrix adhesion and induce detachment of podocytes [18].

Both the tumor suppressor protein p53 and caspases are involved in the crucial processes of renal and nonrenal cell apoptosis [19,20]. The occurrence of podocyte apoptosis directly or indirectly has a correlation with the activation of caspase3, and increased level of p53 induces apoptosis [21,22].

In the present study, it was hypothesized that Angptl3 plays a potential role in the PAN-induced podocyte loss by affecting detachment and apoptosis in vitro. Integrin α3β1, ILK, p53 and caspase3 are molecules of interest for further study to know the possible mechanism. However, the partial result obtained in the present study – recombinant Angptl3 protein aggravated the PAN-induced podocyte detachment is contrary to the results of our previous study, i.e. ANGPTL3 gene transfection ameliorates PAN-induced podocyte detachment [9].

## Methods

### Antibodies and reagents

The antibodies and reagents used in the present study are listed with their sources in parentheses: mouse monoclonal antibody (mAb) to glyceraldehyde-phosphate dehydrogenase (GAPDH), rabbit polyclonal antibody to Angptl3, integrin alpha 3, total integrin beta 1, phospho-integrin beta 1 (phospho T789) and ILK; anti-active + pro- caspase 3 antibody (Abcam, Cambridge, UK); p53 mouse mAb (Cell Signaling Technology, Beverly, USA); recombinant mouse Angptl3 protein (rm-Angptl3) (R&D Systems, Minneapolis, USA); PE Annexin V Apoptosis Detection Kit I (BD Biosciences, San Diego, USA); In Situ Cell Death Detection kit (Roche, Mannheim, Germany); and fluorescein-labelled phalloidin (Sigma Aldrich, St. Louis, USA).

### Cell-lines, culture, and treatment

The mouse podocytes used in this study were a conditionally immortalized cell-line originated by Dr. Peter Mundel (Massachusetts General Hospital, Boston, USA). Culture procedures of the cells were performed according to the standards outlined by Professor Mundel [23]. Briefly, the podocytes were cultured in RPMI 1640 medium containing 10% fetal bovine serum, 100 U/ml penicillin, and 100 ug/ml streptomycin. Podocytes were cultured in a medium containing 10 U/ml mouse interferon-gamma (IFN-γ) at 33°C with 100% relative humidity and a 5% CO2 in air atmosphere to enhance the expression of a thermosensitive T antigen. To induce differentiation, podocytes were grown under non-permissive conditions at 37°C in the absence of IFN-γ for 14 days. At that time, podocytes became large cells with numerous small branches when well differentiated. All cell culture dishes were coated with type I collagen (Sigma- Aldrich). Podocytes were treated with Angptl3 proteins at various doses (50,100,200,250 and 500 ng/ml) for 24 hours, and with Angptl3 proteins (500 ng/ml) for 0, 3, 6, 9, 12, 24, 36 and 48 hours, either alone or after PAN (50 μg/ml, 10 hours) pretreatment. Doses of Angptl3 proteins and times were determined according to our previously reported data [11,24]. All experiments were repeated at least three times.

### Treatment of podocytes with siRNA

Podocytes cultured in six-well plates were incubated with 100 pmol siRNA for 30 hours using Lipofectamine® 2000 (lip2000) Transfection Reagent (Invitrogen, Carlsbad, USA) for transient transfection according to the manufacturer’s protocol of lip2000. Podocytes were 30–50% confluent at the time of transfection. Angptl3-specfic siRNA (Angptl3 siRNA) that was used to silence Angptl3 as well as the control siRNA was designed and produced by Invitrogen. The negative control carboxy-fluorescein - labeled siRNA was used in pilot experiments to determine the optimal transfection rates. The sequences of siRNAs are shown in Table 1.

**Table 1:** **Sequences for all the siRNAs**

			**Sequence**
siRNAs	Control siRNA	sense	UUC UCC GAA CGU GUC AVG UTT
		antisense	ACG UGA CAC GUU CGG AGA ATT
	Angptl3 siRNA	sense	GGG UCA UGG ACU UAA AGA UTT
		antisense	AUC UUU AAG UCC AUG ACC CTT

siRNA, small interfering RNA.

### Western blot analysis

Western blotting was performed according to the standard procedures. An equal amount of each protein lysate was loaded onto 8% or 10% sodium dodecyl sulfate-polyacrylamide gel electrophoresis gels and blotted onto polyvinylidene fluoride membranes. Samples were blocked in Tris-buffered saline Tween 20 (20 mM Tris-HCl, pH 7.4 and 0.05% Tween 20) with 5% non-fat dry milk. The membranes were incubated with primary antibodies at appropriate dilutions overnight at 4°C and horseradish peroxidase-linked secondary antibody (at a dilution of 1:2000) (Santa Cruz, USA) for 1 hour at room temperature. The results were visualized by fluorography using the Tanon gel imaging system (Tanon, Shanghai, China).

### Enzyme linked-immunosorbent assay

After treatment, cell culture supernatant was collected and centrifuged at 1000 g for 20 min to remove particulates and assayed for Angptl3 protein secreted by podocytes by the ANGPTL3 (mouse) enzyme-linked immunosorbent assay (ELISA) Kit (AdipoGen, Liestal, Switzerland) according to the manufacturer’s protocol.

### F-action immunoflurorescence staining

Cells were washed twice with PBS and fixed in 4% paraformaldehyde in PBS for 20 min at room temperature. After the cells were washed three times with PBS, the non-specific binding sites were blocked for 45 min with 3% bovine serum albumin at room temperature before being incubated with fluorescein-labeled phalloidin overnight at 4°C. Nuclei were stained with 4′6′-diamidino-2-phenylindole dihydrochloride (DAPI) for 20 min at room temperature. Images were obtained using an LSM 710 laser scanning confocal microscope (Carl Zeiss, Thornwood, USA). The percentage of stress fibers and mean fluorescence intensity (MFI) of F-actin under confocal microscopy were analyzed as per our previous study by Liu et al. [25]; lamellopodia was quantified as per our previous study by Lin et al. [11]; at least 100 randomly chosen cells were analyzed.

### Detachment assay

The detachment of podocytes was assayed according to our previous study [9]. All cells were cultured on six-well plates under nonpermissive conditions for 14 days prior to the experiments, fields of cells were marked, and cell numbers per field were counted to establish a baseline number. The mean numbers of different fields in three independent sets in this experiment were determined.

### Detection of apoptosis

Both PE-Annexin-V detection of apoptosis and deoxynucleotidyl transferase-mediated dUTP nick-end labeling (TUNEL) assay were used to detect apoptosis. Methods were performed using PE-Annexin-V Apoptosis Detection Kit (BD Biosciences) and In Situ Cell Death Detection kit (Roche) following the manufacturer’s protocol. Overall, 6000 cells per experiment were resuspended with PE-Annexin-V and 7-aminoactinomycin D and analyzed using flow cytometry. TUNEL staining was examined under fluorescence microscope (Olympus, Japan), for each group in given experiments, at least 300 randomly chosen cells were determined for the quantification.

### Statistical analysis

All experiments were repeated at least three times. Data were expressed as mean ± standard deviation. Cell detachment assay was performed by Student’s t-test or one-way analysis of variance followed by the Student Newman-Keuls post hoc test, with *P*-values <0.05 considered statistically significant.

## Results

### Angptl3 affected the PAN-induced podocyte detachment

**Figure 1:**
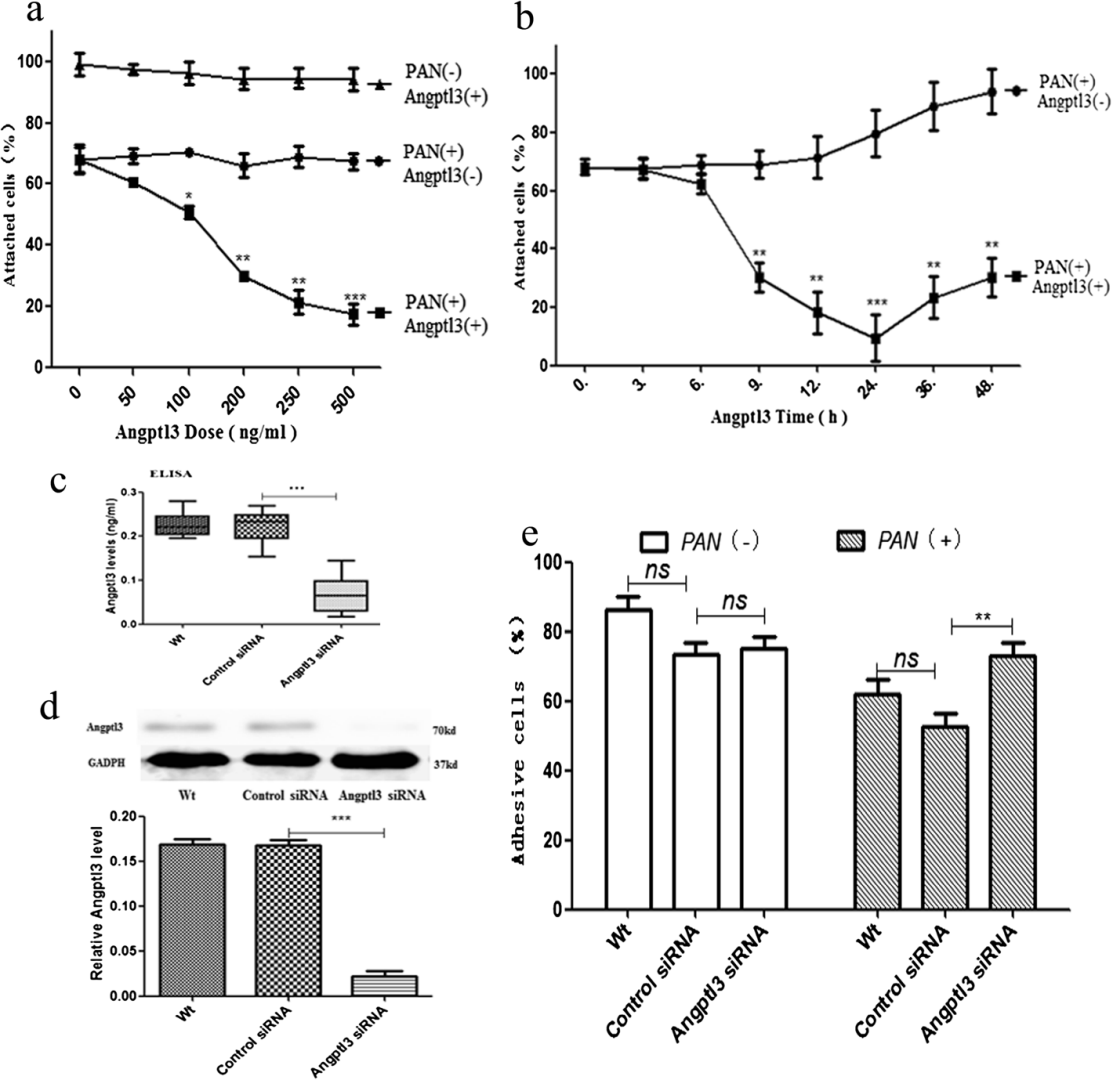
**Angptl3 affected the PAN-induced podocyte detachment. (a)** The detachment was determined in the podocytes at different doses (50,100,200,250 and 500 ng/ml) of rm-Angptl3 with or without PAN (50 μg/ml, 10 hrs) pretreatment for 24 hrs, it shown that rm-Angptl3 without PAN could not change the attached cells in normal podocytes significantly, while, in podocytes pretreated with PAN, rm-Angptl3 reduced the attached cells markedly, and the more the doses (100-500 ng/ml), the less the attached cells. **(b)** The rates of attached cells podocytes treated with rm-Angptl3 for different times after PAN pretreatment; the cells decreased from 9 h to 48 h, and the rate of 24 h was the lowest. **(c)** ELISA results showing weaker secretion of Angptl3 in the medium of Angptl3 siRNA group (lowered by 69.50%); **(d)** Western blot confirmed that Angptl3 was knocked down by siRNA successfully (reduced by 86.89 ± 3.58%). **(e)** There was no statistical difference between cell number in the Wt group and control siRNA group with or without PAN pretreatment. Cell number in the control siRNA group and Angptl3 siRNA group were almost the same, while, after PAN pretreatment, cell number in the Angptl3 siRNA group is more than in the control siRNA group. (Wt: wide type; Control siRNA: podocytes transfected with control siRNA; Angptl3 siRNA: podocytes transfected with Angptl3 siRNA. *P <0.05, **P <0.01, ***P <0.0001) Angptl3, angiopoietin-like3; ELISA, enzyme-linked immunosorbent assay; PAN, puromycin aminonucleoside; siRNA, small interfering RNA.

The effect of Angptl3 on tpodocyte detachment was evaluated in podocytes treated with exogenous rm-Angptl3 and Angptl3 siRNA. There was no significant difference in the rate of attached cells in podocytes treated with different doses of rm-Angptl3 alone during 24 hours. However, a significant difference was noted in the the rate of attached cells of podocytes which treated with rm-Angptl3 (100-500 ng/ml, 24 h) in combination with PAN pretreatment. The rm-Angptl3 markedly accelerated the PAN-induced podocyte detachment, and in a dose-dependent manner (Figure 1a). It was also found that rm-Angptl3 (500 ng/ml) initiated the aggravation of PAN-induced detachment of podocyte at 9 h, and reached a maximum at 24 h (Figure 1b).

Angptl3 siRNA was used to inhibit the expression of Angptl3 in podocytes. Compared to control siRNA, Angptl3 siRNA resulted in a highly significant reduction in the expression and secretion of Angptl3 protein (Figure 1c and d). The knockdown of Angptl3 by Angptl3 siRNA rarely influenced the detachment of podocytes under normal conditions, while Angptl3 siRNA markedly rescued the detachment of podocytes induced by PAN (Figure 1e).

### Apoptosis almost occurred simultaneously with detachment in Angptl3-influenced podocyte loss

**Figure 2:**
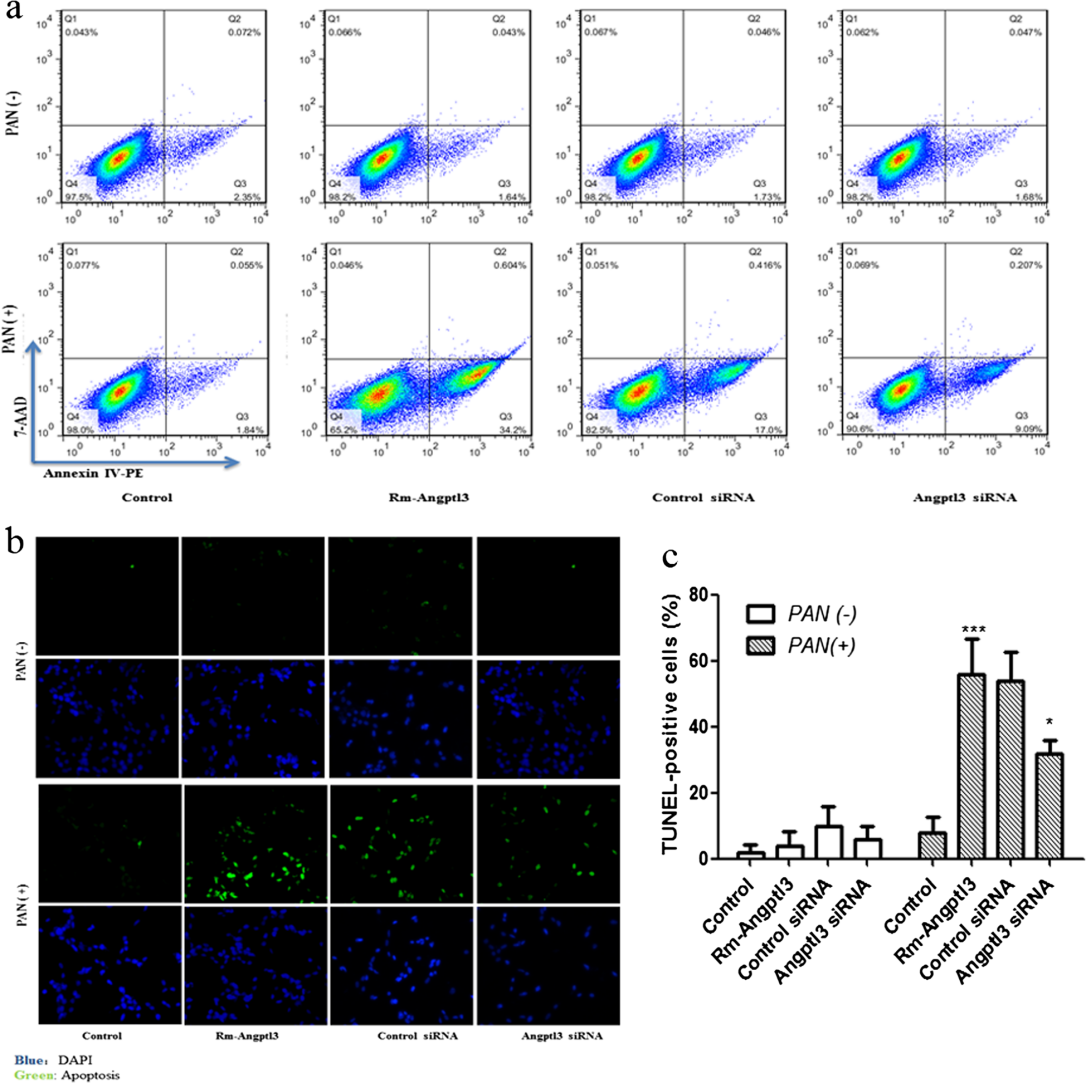
**Angptl3 affected apoptosis in PAN-induced podocyte injury model. (a)** Flow cytometry analysis of the rate of apoptosis in the cultured podocytes with different treatments. No significant differences in the number of apoptotic cells were observed among control, rm-Angptl3, control siRNA and Angptl3 siRNA groups. After PAN pretreatment, the rate of apoptosis was significantly increased in the rm-Angptl3 group compared to the control group (34.80±7.9 vs. 1.89±0.92 3%, *P*<0.0001; n=6). And in comparison to the control siRNA group, apoptosis rate in the podocytes treated with Angptl3 siRNA was a little lower (9.29±1.27vs. 17.42±2.61%, *P*<0.05; n=6). **(b, c)** This finding was confirmed using TUNEL assay for apoptosis in cultured podocytes. In PAN-induced podocyte injury model, TUNEL positive cells in rm-Angptl3-treating group are significantly more than that without rm-Angptl3 treating (56±9 vs. 8±10 %, *P*<0.0001; n=6), and TUNEL positive cells in the Angptl3 siRNA group are significantly less than that in control siRNA group (32±4 vs. 54±9 %, *P*<0.05; n=6) (magnification ×400). (Control: podocytes treated without rm-ANGPTL3; Rm-ANGPTL3: podocytes treated with rm-ANGPTL3. *P<0.05, ***P<0.0001). Angptl3, angiopoietin-like3; PAN, puromycin aminonucleoside; siRNA, small interfering RNA; TUNEL. deoxynucleotidyl transferase-mediated dUTP nick-end labeling.

Podocyte apoptosis was detected simultaneously with detachment assay under the same experimental conditions. The flow cytometry analysis and TUNEL assay showed that rm-Angptl3 or Angptl3 siRNA alone could not induce the apoptosis of podocytes under normal conditions (Figure 2). The rate of apoptosis detected by flow cytometry in the podocytes with rm-Angptl3 combined with PAN pretreatment was significantly higher than that in podocytes treated with PAN alone. When the podocytes were pretreated with PAN, the rate of apoptosis in the Angptl3 siRNA group was a little lower than that in the control siRNA group (Figure 2a). A similar finding was obtained using the TUNEL assay for apoptosis in cultured podocytes (Figure 2b and c).

### F-actin rearrangement was involved in Angptl3-influenced podocyte loss

**Figure 3:**
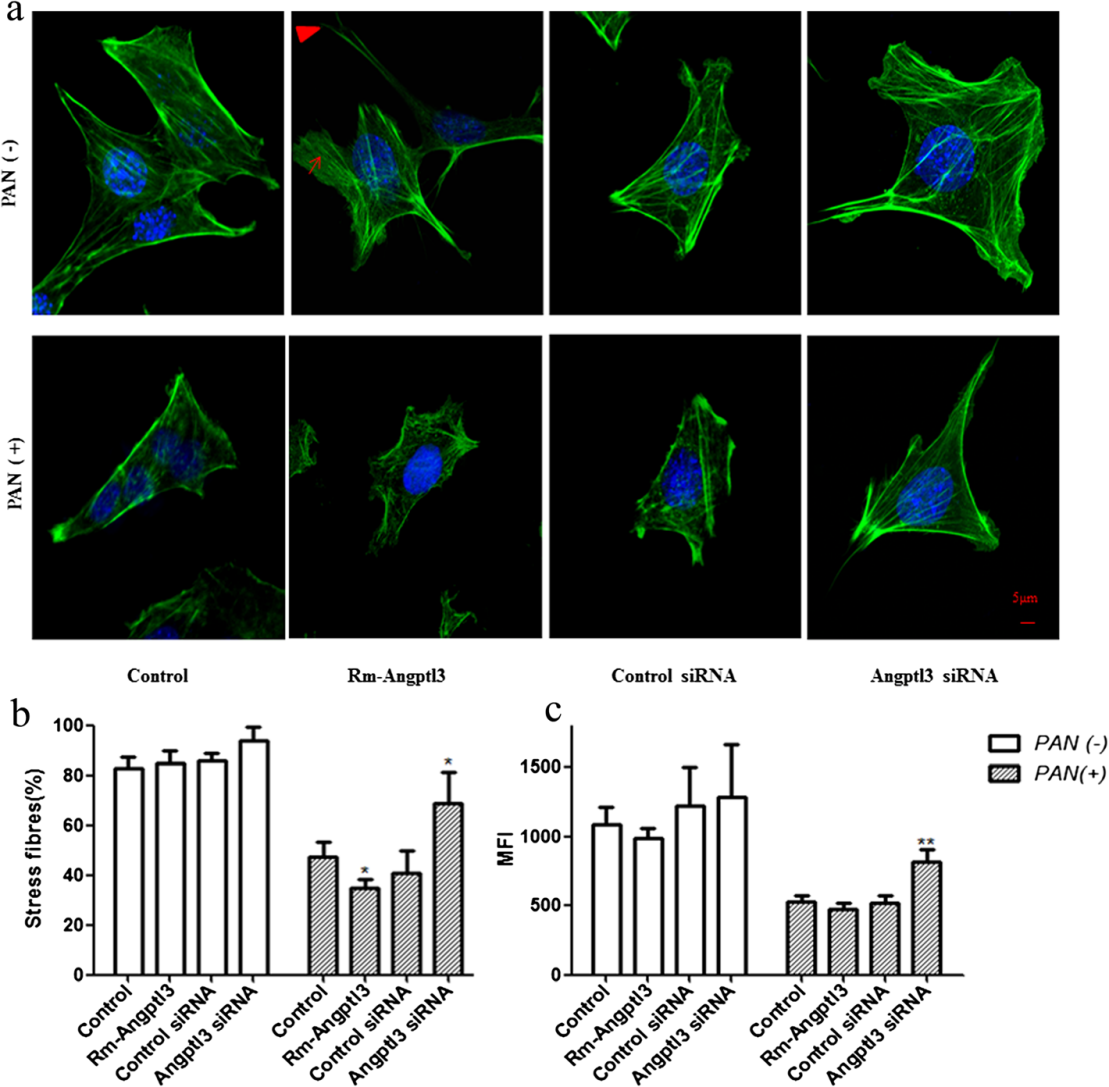
**Angptl3 influenced the F-actin rearrangement in podocytes. (a)** Phalloidin fluorescence labelling of F-actin showed podocyte stress fibers. Confocal microscopy showed no difference in the F-actin staining pattern between control group and Angptl3 siRNA group. However, F-actin rearrangement in Angptl3 group, characterized by the formation of lamellipodia (arrowhead) and increase in cell spikes (arrow). In PAN induced podocyte injury model,cells became retracted and F-actin seemed partial disrupted,moreover, F-actin almost completely disrupted in Angptl3 protein group. However, disruption did not occur in Angptl3 knockdown group. **(b)** The percentage of stress fibers also showed that Angptl3 protein could significantly aggravate the effect of PAN on decreaseing podocyte stress fibers while Angptl3 siRNA could prevent PAN from decreasing podocyte stress fibers. **(c)** Quantitative analysis showed the MFI of F-actin in different groups under confocal microscopy. After PAN treatment, MFI value in the Angptl3 siRNA group is higher than in the control siRNA group. (Scale bar, 5 μm *P <0.05, **P <0.01. n=100). Angptl3, angiopoietin-like3; MFI, mean fluorescence intensity; PAN, puromycin-aminonucleoside; siRNA, small interfering RNA.

Angptl3 influenced the detachment and apoptosis in the PAN-induced podocyte loss. To further investigate whether F-actin rearrangement is involved in the observed process, fluorescent phalloidin-labeled F-actin was used to observe actin cytoskeletal rearrangement in podocytes. As shown in Figure 3, podocytes treated with rm-Angptl3 formed more spikes and lamellipodia compared to the transversal F-actin location in control podocytes. There were no significant differences in the F-actin staining pattern between podocytes transfected with Angptl3 siRNA and control siRNA. When the podocytes were pretreated with PAN, the disruption of F-actin filament was found to be more severe in podotyes treated with rm-Angptl3 compared to the podocytes without treatment with rm-Angptl3, while the Angptl3 siRNA ameliorated the PAN-induced disruption of F-actin filament (Figure 3a).

The present data on stress fibers and MFI of F-actin demonstrated that rm-Angptl3 in combination with PAN pretreatment significantly disrupted stress fiber, while, administration of PAN failed to decrease stress fiber contents in the Angptl3 knockdown podocytes (Figure 3b). The MFI of F-actin further showed that Angptl3 siRNA alleviated the reduction in MFI induced by PAN (Figure 3c).

### Integrin α3β1, ILK, and p53, rather than caspase 3, are necessary for Angptl3 to affect the PAN-induced podocyte loss

To elucidate the possible molecular mechanism of Angptl3 on PAN-induced podocyte loss, the expression of integrin α3, total integrin β1, phospho-integrin β1, ILK, p53, and caspase 3 was examined in cultured podocytes with various treatments.

**Figure 4:**
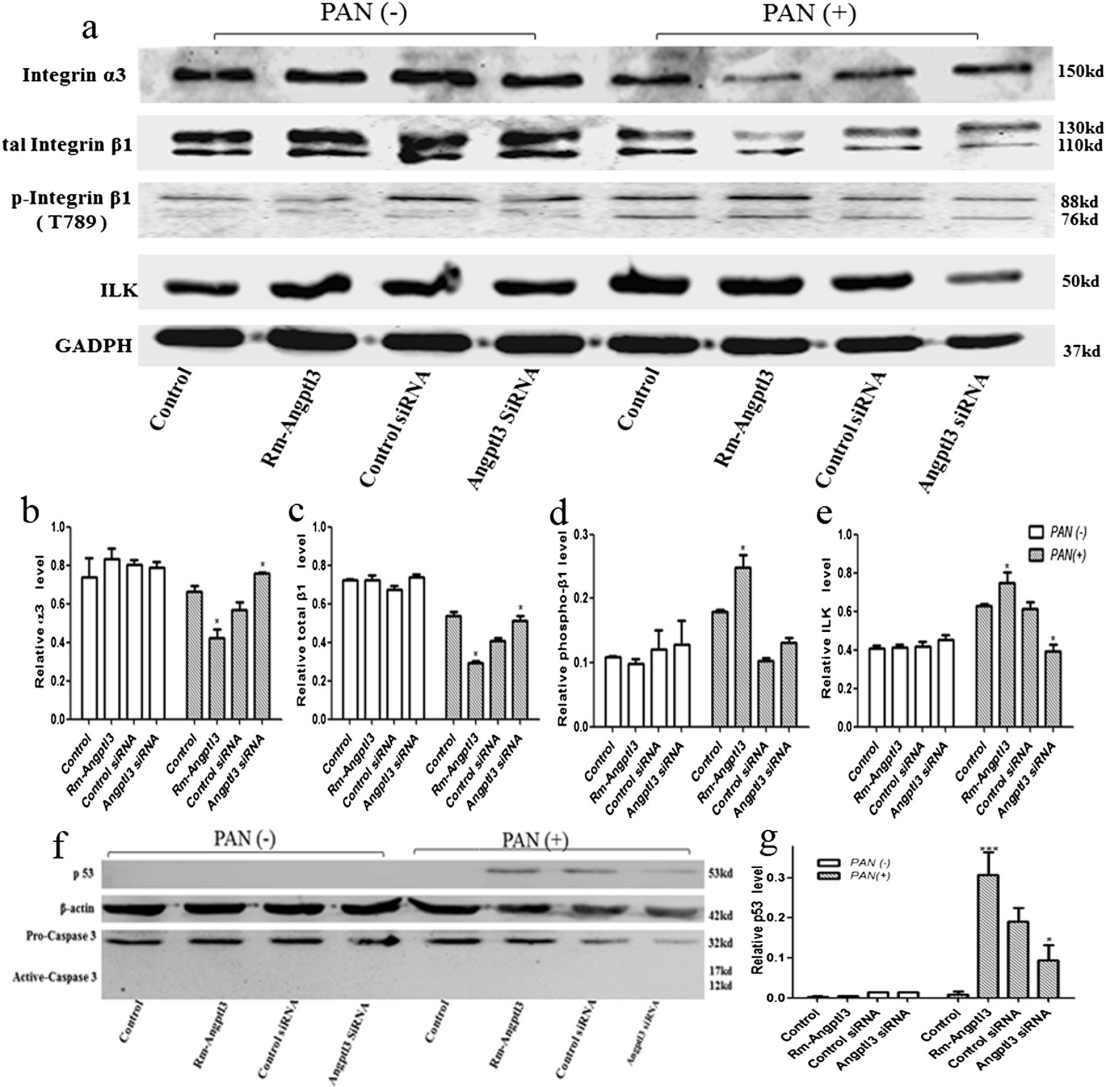
**Angptl3 orchestrated integrin α3β1, ILK and p53, rather than caspase 3 in podocytes on treatment with PAN. (a)** Western blots were performed on the expression of integrin α3, total integrin β1, phospho-integrinβ1, and ILK in podocytes with different treatment, it was showed that, there was no significant difference of in the observed molecular expression in podocytes without PAN pretreatment, while, after treatment with PAN, expression of integrin α3 and total integrin β1 became lower (ILK and phospho- integrin β1, higher) in the rm-Angptl3 group than that in the control group, and Angptl3 siRNA group showed higher expression of integrin α3, total integrin β1 (ILK, lower) than in control siRNA group, while, phospho- integrinβ1 in the two group had no statistical difference. **(b, c, d, e)** Relative levels of integrin α3, total integrin β1, phospho- integrinβ1 and ILK. **(f, g)** Western blots suggesting that there was rarely no activated caspase 3 in any observed groups; with PAN pretreatment, p53 protein level was markedly increased in rm-Angptl3 group with comparison to the control group, while, p53 protein level in Angptl3 siRNA group was a little lower than that in the control siRNA group, no p53 protein was detected in any other groups. (*P<0.05, **P<0.01, ***P<0.0001 n=6). Angptl3, angiopoietin-like3; ILK, integrin-linked kinase; PAN, puromycin aminonucleoside; siRNA, small interfering RNA.

Western blots confirmed lower expression of integrinβ1 and overexpression of phospho-integrin β1and ILK in the PAN induced podocyte injury model in vitro [18,20]. Angptl3 could not alter the expression of integrin α3, total integrin β1, phospho-integrin β1, and ILK in podocytes under normal conditions. However, rm-Angptl3 and Angptl3 siRNA displayed remarkable changes in the levels of integrin α3, total integrin β1, phospho-integrin β1 and ILK in podocytes with PAN pretreatment: rm-Angptl3 weakened the expression of integrin α3β1 and strengthened the phospho-integrin β1 and ILK; on the contrary, the knockdown of Angptl3 by siRNA prevented weakening of integrin α3β1, and enhancement of ILK in PAN-induced podocyte detachment, but no difference in the levels of phospho-integrin β1 between the Angptl3 siRNA and control groups (Figure 4a-e). Rm-Angptl3 combined with PAN markedly increased p53 protein levels, while, in PAN-induced podocyte injury model, p53 protein levels in the Angptl3 siRNA group was a little lower than that in the control siRNA group. No increased activation of caspase 3 was observed in any groups of podocytes in the present study (Figure 4f-g).

## Discussion

Progressive glomerulosclerosis accounts for the vast majority of chronic kidney disease leading to end-stage renal disease [26]. Ongoing podocyte loss contributes to the progression of glomerulosclerosis and proteinuria, as podocytes cannot be replaced easily [2,27]. PAN model is one of the best described models of podocyte injury, which could induce cytoskeletal disaggregation, detachment and apoptosis in podocytes [16,28,29].

First, the present study indicated that Angptl3 played a vital role in the PAN-induced podocyte loss, both detachment and apoptosis occurred, and F-actin rearrangement was involved in the process of Angptl3-influenced podocyte loss.

It was found that rm-Angptl3 could not induce the detachment of podocytes under normal conditions. Interestingly, in the PAN-induced podocyte injury model, rm-Angptl3 aggravated the detachment, and the knockdown of Angptl3 served to rescue detachment. Curiously, this partial finding in the present study – rm-Angptl3 aggravated the PAN-induced podocyte detachment was in contrast to our previous study by Rao et al. – ANGPTL3 gene transfection ameliorates the PAN-induced podocyte detachment [9]. The experimental methods and conditions in the two studies were different: in the former study, recombinant Angptl3 protein was used to treat podocytes to mimic the conditions of Angptl3 overexpression, most of the Angptl3 protein found in the culture medium was derived from the exogenous recombinant Angptl3 protein, weakly secreted by podocytes; in the latter study, recombinant plasmid pcDNA3.1-ANGPTL3 was transfected into podocytes with lip2000 reagent to promote the overexpression of Angptl3, the Angptl3 protein presented in culture medium was all secreted by podocytes. Angptl3 is a secreted glycoprotein, Ge et al. performed that Angptl3 can form higher-order structures [30], and Ryuta, et al. suggested deglycosylation reduces the apparent molecular mass of the secreted recombinant Angptl3 (70kD) to 53 kD [31]. We detected the podocyte Angptl3 proteins and the exogenous recombinant Angptl3 proteins by western blotting with anti- Angptl3 polyclonal antibody, both protein bands of approximately 70kD and 53kD appeared in the podocytes Angptl3 proteins, while, only protein band of 70kD (no 53kD protein band) appeared in the recombinant Angptl3 proteins (data not shown). We therefore suggest that the podocyte-specific Angptl3 and the exogenous recombinant Angptl3 might have different protein modifications and/or structures. Clement et al. found podocyte-specific Angptl4 and circulating Angptl4 might play different roles in proteinuria [32,33]. Angptl3 is a homolog of Angptl4 [30]. Hence it was speculated that the different experimental methods and conditions might partially influence the contrasting results; moreover, much of the contrasting results is likely due to the differential roles the podocyte-specific Angptl3 and the exogenous Angptl3 proteins played in podocyte detachment, and the differential roles might result from the different protein modifications and/or structures. The contradictory results indicate that exogenous recombinant Angptl3 proteins may be a damaging factor in PAN-induced podocyte detachment, while, podocyte-specific Angptl3 may provide a protective role to some extent under abnormal states. However, direct evidence is lacking, further studies are needed to confirm the present results.

Apoptosis is one of the key factors in determining the number of podocytes in the glomeruli [3]. And it was found in this study that rm-Angptl3 combined with PAN could induce podocyte apoptosis, while the knockdown of Angptl3 ameliorated apoptosis of podocytes with PAN pretreatment. So we concluded that Angptl3 affected apoptosis almost simultaneously with detachment in PAN-induced podocyte injury model, both detachment and apoptosis occurred in Angptl3-influenced podocyte loss.

Using confocal microscopy to assess the rearrangement of F-actin in podocyte under the same experimental conditions in our present study, it was confirmed that Angptl3 induces the rearrangement of actin filament in podocytes, which is consistent with our previous observation [13]. Moreover, knockdown of Angptl3 by siRNA failed to change F-actin filament, which may indicate that Angptl3 is not a necessary conditional molecule in maintaining the intricate shape and structure of podocytes. Rm-Angptl3 combined with PAN pretreatment disrupted F-actin filament, knockdown of Angptl3 attenuated the rearrangement of F-actin induced by PAN. It was confirmed that F-actin rearrangement was involved in the process of Angptl3-influenced podocyte loss. The results of the present study indicated that decrease or disruption of podocytes stress fibers may aggravate the loss of podocytes.

Second, the present study pointed to the possible molecular mechanism of the observed effects of Angptl3. Both integrin α3β1 and ILK are key molecules in podocyte detachment, ILK is in its inactive state under normal resting conditions, PAN-induced podocyte damage could activate ILK, and active ILK can phosphorylate the cytoplasmic domain of β1-integrin, which results in a low-affinity binding state and podocytes are detached from the GBM[15-18]. It is likely that the combined action of both integrin α3β1 and ILK accounts for the effects of Angptl3 on PAN-induced podocyte detachment, which were partially correlated with Angptl3 could affect the influence of PAN on altering the expression of integrin α3, total integrinβ1, phosphorylation of integrin β1 and ILK. Angptl3 may affect the PAN-induced detachment via ILK/integrin α3β1 pathway, detection of the activity of ILK and observation of the results after inhibiting ILK, which would provide much more information.

Changes in activated caspase 3 and increased levels of p53 are often observed in podocyte apoptosis [21]. In the present study, it was found that the increased p53 was positively correlated with the increased apoptosis, while activated caspase 3 was rarely observed. Mohr, et al. found that not all apoptosis requires caspase-3 activation [34], so we speculated that the alterations of p53 protein levels rather than activation of caspase3 contributed to the influence of ANGPTL3 on apoptosis in the present study. Taken together, the effects of Angptl3 may be more complex than currently understood, and the exact mechanism needs to be further studied.

## Conclusion

Angptl3 is a novel factor involved in PAN-induced podocyte loss by affecting detachment and apoptosis. The present study might be helpful in understanding the mechanisms of podocyte loss, and in developing a new successful therapy for podocyte injury via lower expression of Angptl3.

## Abbreviations

Angptl3: Angiopoietin-like3
rm-Angptl3: Recombinant mice Angptl3 protein
siRNA: Small interfering RNA
ILK: Integrin-linked kinase
PAN: Puromycin aminonucleoside
GBM: Glomerular basement membrane
Angptl4: Angiopoietin-like-4
GAPDH: Glyceraldehyde-phosphate dehydrogenase
lip2000: Lipofectamine®2000 Transfection Reagent
ELISA: Enzyme-linked immunosorbent assay
TUNEL: Transferase-mediated dUTP nick-end labeling
MFI: Mean fluorescence intensity
